# Gestational weight gain as a risk factor for dystocia during first delivery: a multicenter retrospective cohort study in Japan

**DOI:** 10.1186/s12884-022-05055-6

**Published:** 2022-09-23

**Authors:** Hyo Kyozuka, Tsuyoshi Hiraiwa, Tsuyoshi Murata, Misa Sugeno, Toki Jin, Fumihito Ito, Daisuke Suzuki, Yasuhisa Nomura, Toma Fukuda, Shun Yasuda, Keiya Fujimori

**Affiliations:** 1 Department of Obstetrics and Gynecology, Ohta Nishinouchi Hospital, 2-5-20, Nishinouchi, Koriyama city, Fukushima 963-8558 Japan; 2 Department of Obstetrics and Gynecology, Fukushima Medical University School of Medicine, 1 Hikarigaoka, Fukushima, 960-1295 Japan; 3 Department of Obstetrics and Gynecology, Iwase General Hospital, 20, Kitamachi, Sukagawa city, Fukushima 962-8503 Japan; 4 Department of Obstetrics and Gynecology, Shirakawa Kosei General Hospital, Fukushima, 961-0005 Japan

**Keywords:** Gestational weight gain, Dystocia, Nullipara, Preconception care, Obesity

## Abstract

**Background:**

Dystocia is a common obstetric complication among nulliparous women, which requires medical intervention and carries the risk of negative maternal and neonatal outcomes. Our aim was to examine the association between body mass index (BMI) and the occurrence of dystocia. We also identified cutoffs of gestational weight gain, based on pre-pregnancy BMI, associated with the risk of dystocia.

**Methods:**

This was a multicenter, retrospective, cohort study conducted in two tertiary Maternal–Fetal medicine units in Fukushima, Japan. The study population included nullipara women who delivered at either of the two units between January 1, 2013, and December 31, 2020. Women (*n* = 2597) were categorized into six groups (G) based on their pre-pregnancy BMI: G1 (< 18.5 kg/m^2^), G2 (18.5 to < 20.0 kg/m^2^), G3 (20.0 to < 23.0 kg/m^2^), G4 (23.0 to < 25.0 kg/m^2^), G5 (25.0 to < 30.0 kg/m^2^), and G6 (≥ 30.0 kg/m^2^). Using G3 as a reference, multiple logistic regression analyses were performed to estimate the risk of dystocia for each BMI category. Receiver operating characteristic curve analyses were performed to determine the cutoff value of gestational weight gain for the risk of dystocia.

**Results:**

The highest BMI category (G6) was an independent risk factor for dystocia (adjusted odds ratio, 3.0; 95% confidence interval, 1.5–5.8). The receiver operating characteristic curve analysis revealed no association between gestational weight gain and the occurrence of dystocia in G5 and G6 (*P* = 0.446 and *P* = 0.291, respectively). For G1 to G4, AUC and predictive cutoffs of gestational weight gain for dystocia were as follows: G1, AUC 0.64 and cutoff 11.5 kg (*P* < 0.05); G2, AUC 0.63 and cutoff 12.3 kg (*P* < 0.05); G3, AUC 0.67 and cutoff 14.3 kg (*P* < 0.01); and G4, AUC 0.63 and cutoff 11.5 kg (*P* < 0.05).

**Conclusion:**

A pre-pregnancy BMI > 30.0 kg/m^2^ was an independent risk factor for dystocia. For women with a pre-pregnancy BMI < 25.0 kg/m^2^, the risk of dystocia increases as a function of gestational weight gain. These findings could inform personalized preconception care for women to optimize maternal and neonatal health.

## Background

Dystocia, defined as an abnormality in the progression of labor, is one of the most common obstetric complications among nulliparous women for which medical intervention is justified [1, 2]. Identifying the risk of dystocia is essential as dystocia is associated with operative vaginal delivery, cesarean section (CS), and postpartum hemorrhage [3, 4]. In addition to being a maternal health risk, dystocia also increases the risk of intrauterine and fetal inflammation, which can have a negative impact on the neurodevelopment of neonates [5, 6].

Obesity during pregnancy has become a global health problem [7]. Dystocia is associated with obstetric issues, particularly among nullipara women who have a high pre-pregnancy body mass index (BMI) [7–9]. Although the underlying pathophysiology of dystocia remains unclear, a high maternal BMI has been associated with a higher frequency of a prolonged first stage of labor [8, 9]. In Japan, obesity in pregnant women is defined as a pre-pregnancy BMI ≥ 25.0 kg/m^2^ [10]. Generally, BMI is categorized into the following five validated groups (G): G1, < 18.5 kg/m^2^; G2, 18.5 to < 20.0 kg/m^2^; G3, 20.0 to < 23.0 kg/m^2^; G4, 23.0 to < 25.0 kg/m^2^; G5, 25.0 to < 30.0 kg/m^2^ (overweight); and G6, ≥ 30.0 kg/m^2^ (obesity) [7, 11–15].

Recently in Japan, gestational weight gain, based on maternal pre-pregnancy BMI, has been recognized as an important modifiable determinant of pregnancy outcomes [11–15]. While an association between gestational weight gain and adverse perinatal outcomes has previously been reported [11–15], the association between gestational weight gain and dystocia as a function of pre-pregnancy BMI, based on the six BMI groups, remains to be clarified. Accordingly, our aim in this study was to examine the association between BMI and the occurrence of dystocia among nulliparous women in Japan, as well as to identify the appropriate gestational weight gain cutoffs, based on the pre-pregnancy BMI, to lower the risk of dystocia.

## Methods

### Study patients

This was a multicenter retrospective cohort study conducted in two tertiary Maternal–Fetal medicine units in the Fukushima Prefecture, Japan. The study population consisted of pregnant women who delivered at either of these two units between January 1, 2013, and December 31, 2020. Multiparous cases, cases with insufficient data, and cases in which delivery occurred before 22 weeks were excluded.

### Statement of ethics

Our study was approved by the institutional review board (IRB) of Ohta Nishinouchi Hospital (No. 37). The requirement for informed consent was waived owing to the retrospective design of the study and use of anonymized data by the IRB of Ohta Nishinouchi Hospital (No. 37). All procedures performed in this study were in accordance with the ethical standards of our institutional and/or national research committee and the 1964 Helsinki Declaration and its later amendments or comparable ethical standards.

### Maternal and neonatal information

Maternal and neonatal information was extracted from the medical records of the women included in the study sample at each medical unit. The following maternal information was collected: age at delivery, parity, maternal height, weight before pregnancy and at delivery, methods of conception and delivery, gestational weight gain, maternal smoking, and presence of uterine myoma. Neonatal information included gestational age at delivery and birth weight. As maternal age is not linearly associated with maternal complications [16], maternal age at delivery was categorized into the following four groups for analysis: < 20, 20–29, 30–39, and ≥ 40 years. The method of conception was dichotomized as presence or absence of assisted reproductive technology (ART) pregnancy, where ART pregnancy was defined as conception after in vitro fertilization and intracytoplasmic sperm injection or cryopreserved, frozen, or blastocyst embryo transfers [17]. Maternal BMI was calculated according to World Health Organization standards (body weight [kg] / height^2^ [m^2^], kg/m^2^). Participants were categorized into six groups (G) for analysis, according to their pre-pregnancy BMI: G1, < 18.5 kg/m^2^; G2, 18.5 to < 20.0 kg/m^2^; G3, 20.0 to < 23.0 kg/m^2^; G4, 23.0 to < 25.0 kg/m^2^; G5 (25.0 to < 30.0 kg/m^2^), and G6 (≥ 30.0 kg/m^2^).

### Dystocia and other maternal complications

Dystocia is described as a cephalic presentation for delivery in a woman, requiring either (1) operative vaginal delivery with several trials of maternal effort due to arrest of the active phase of labor for at least 4 h, when cervical dilation is 10 cm, with or without the use of an augmentation agent or (2) no progression of cervical dilation despite clinically adequate effective labor, which is defined as one contraction every 10 min, irrespective of augmentation, such as the use of oxytocin or performance of amniotomy [5, 18].

Birth weight was measured by the midwife immediately after delivery. The z-scores of birth weight were calculated using the “New Japanese Neonatal Anthropometric Charts” [19–21]. Small for gestation age (SGA) was defined as a birth weight less than − 1.5 SD below the population mean appropriate for gestational age [11, 20]. Hypertensive disorders of pregnancy (HDP) was defined as new-onset hypertension after 20 weeks of gestation or presence of chronic hypertension before pregnancy [22]. New-onset hypertension after 20 weeks was further categorized into preeclampsia (PE) and gestational hypertension (GH). Phenotype PE and GH is defined with or without proteinuria, SGA, and/or liver dysfunction [22]. All pregnant women included in our study sample had undergone screening for gestational diabetes mellitus (GDM), both in early and late pregnancy [23]. In Japan, glucose tolerance screening and testing for GDM are performed for every pregnant woman, according to the protocols recommended by the Obstetrics Society and Diabetes Society of Japan; depending on the local obstetrics institution, a two-step protocol is followed during both first and second or third trimesters [23]. The diagnostic criteria for GDM is reported elsewhere [24, 25].

### Statistical analyses

First, we compared the maternal background and obstetric outcomes between women with and without dystocia using Student’s t-test for continuous variables and the chi-squared test for categorical variables. Obstetric outcomes were then compared between BMI categories to determine differences in occurrence of outcomes between BMI groups, using Jonckheere’s trend test and the extended Mantel–Haenszel chi-squared test for linear trends for continuous and categorical variables, respectively. To evaluate the association between BMI and the risk of dystocia, the odds ratios (ORs) and 95% confidence intervals (CIs) for dystocia using logistic regression analysis were calculated. In Model 1, univariate analysis was conducted using the G3 BMI as the reference. In Model 2, multivariate regression was performed controlling for the following independent variables and calculating the adjusted ORs (aORs) and 95% CIs for dystocia, again using G3 BMI as the reference: chronic HT, maternal smoking status, ART pregnancy, maternal age (20–29 years as reference), and myoma uteri. In Model 3, GDM was added to the variables controlled for in Model 2 and, again, the aOR and 95% CI were calculated. As weight gain during pregnancy is a continuous variable, a receiver operating characteristic curve (ROC) was constructed to predict the probability of dystocia due to weight gain during pregnancy. Area under the curve (AUC) analysis was performed to calculate the diagnostic ability of weight gain during pregnancy for dystocia and to determine the cutoffs of gestational weight gain associated with dystocia. All statistical analyses were performed using SPSS (v26; IBM Corp., Armonk, NY, USA), with a *P-*value of < 0.05 indicating statistical significance.

## Results

**Fig. 1:**
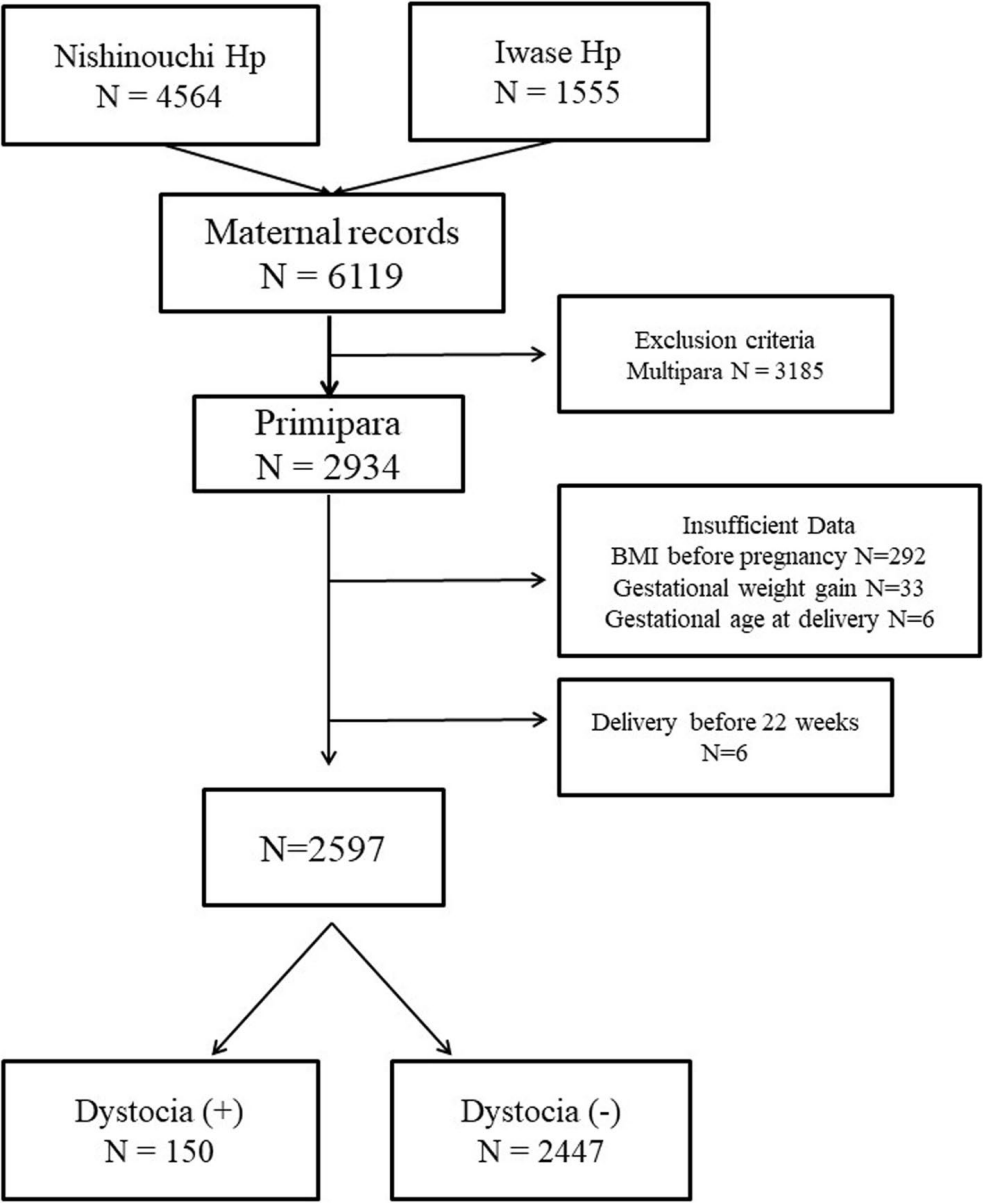
Flowchart of the selection of pregnant women for the study sample

Figure 1 shows the flowchart for the selection of pregnant women for the study sample. During the study period, there were 4,564 and 1,555 singleton deliveries at the Ohta Nishinouchi Hospital and Iwase Hospital, respectively. Among these, 3,185 were excluded because of multiparity, 331 due to insufficient available data, and 6 due to delivery before 22 weeks of gestation. After selection, 150 cases were classified in the “with” dystocia group and 2,447 in the “without” dystocia group.

The maternal background characteristics and obstetric outcomes for the with and without dystocia groups are reported in Table 1. Of these background maternal characteristics, the mean maternal age (*P* = 0.024), presence of uterine myoma (*P* = 0.042), ratio of ART to no ART pregnancy (*P* = 0.013), mean pre-pregnancy BMI (*P* < 0.001), and mean maternal weight at delivery (*P* < 0.001) were higher in the with dystocia than in the without dystocia group. Regarding neonatal characteristics, mean gestational weeks at delivery, mean birth weight, and SD of birth weight were significantly higher in the with dystocia group (all < 0.001). SGA was significantly higher in the without dystocia (9.6%) than in the with dystocia (4.7%) group (*P* = 0.048). The proportion of CS was higher in the with dystocia (76.7%) than in the without dystocia (15.8%) group (*P* < 0.001).

**Table 1:** Maternal characteristics and obstetric outcomes based on the “with” or “without” dystocia criterion

Variable	Study Sample		*P*-value
	Without Dystocia	With Dystocia	
	*n* = 2447	*n* = 150	
Maternal Background			
Age, years, mean (SD)	29.7 (5.6)	30.8 (5.6)	0.024^a^
% in each age category, years			
≤ 19	3.6	1.4	0.151^b^
20–29	46.2	42.8	
30–39	45.3	47.6	
≥ 40	5.0	8.3	
Smoking during pregnancy, %	8.8	12.2	0.167^b^
Uterine myoma, %	4.9	8.7	0.042^b^
ART pregnancy, %	6.2	11.3	0.013^b^
BMI before pregnancy, kg/m^2^, mean (SD)	21.9 (4.0)	23.9 (6.0)	< 0.001^a^
% in each BMI category, kg/m^2^			
< 18.5	14.1	12.7	< 0.001^b^
18.5 to < 20.0	20.8	18.0	
20.0 to < 22.9	37.5	25.3	
23.0 to < 25.0	11.6	12.0	
25.0 to < 30.0	10.7	16.7	
≥ 30.0	5.4	15.3	
Gestational weight gain, kg, mean (SD)	9.5 (5.0)	10.3 (4.8)	0.072^a^
Weight at delivery, kg, mean (SD)	64.3 (10.3)	69.5 (14.1)	< 0.001^a^
Obstetric outcomes			
Gestational age at delivery, weeks, mean (SD)	38.5 (2.5)	39.7 (1.2)	< 0.001^a^
Birthweight, g, mean (SD)	2836 (547)	3225 (409)	< 0.001^a^
SD of birthweight, mean (SD)	-0.14 (1.07)	0.46 (1.09)	< 0.001^a^
SGA, %	9.6	4.7	0.048^b^
HDPs, %	9.6	14.7	0.046^b^
GDM, %	4.9	6.7	0.350^b^
CS, %	15.8	76.7	< 0.001^b^

*SD* Standard deviation, *ART* Assisted reproductive technology, *BMI* Body mass index, *SGA* Small for gestational age, *HDPs* Hypertension disorders of pregnancy, *GDM* Gestational diabetes mellitus, *CS* Cesarean section, *BMI* Body mass index

^a^*p* value, Student’s t-test

^b^*p* value, chi-squared test

Obstetric outcomes for each BMI category are reported in Table 2. The occurrence of HDPs, PE, GH, and GDM significantly increased as a function of increasing BMI category (all *P* < 0.001). Jonckheere’s trend test revealed that maternal birth weight increased as a function of increasing BMI category (*P* < 0·001), while gestational weight gain decreased (*P* < 0·001). The occurrence of SGA and dystocia showed no significant trend (*p* = 0.386 and *p* = 0.165, respectively). The occurrence of dystocia was lowest in the G3 group (4.0%) and highest in the G6 group (14.9%).

**Table 2:** Trend in obstetrics outcome as a function of the body mass index category

BMI category (kg/m^2^)	< 18.5 (G1)	18.5 to < 20.0 (G2)	20.0 to < 23.0 (G3)	23.0 to < 25.0 (G4)	25.0 to < 30.0 (G5)	≥ 30.0 (G6)	*P*-value
Number of patients	365	535	953	301	286	154	
Gestational weight gain, kg, median (IQR)	10.5 (8.1–13.0)	10.3 (8.0–12.4)	10.1 (7.8–12.9)	9.9 (6.8–12.9)	7.9 (5.0–11.7)	4.4 (0.5–8.0)	< 0.001^a^
Birthweight, g, median (IQR)	2840 (2535–3089)	2885 (2640–3145)	2924 (2630–3185)	2940 (2675–3285)	2997 (2665–3286)	3023 (2629–3287)	< 0.001^a^
SGA, %	11.0	10.1	8.7	8.0	9.4	11.0	0.386^b^
HDPs, %	6.3	5.4	8.0	12.6	16.4	29.2	< 0.001^b^
PE, %	4.4	3.5	5.3	7.3	7.7	13.6	< 0.001^b^
GH, %	1.9	1.5	2.2	4.3	6.3	10.4	< 0.001^b^
GDM, %	3.8	3.0	3.1	8.0	8.7	14.3	< 0.001^b^
Dystocia, %	5.2	5.0	4.0	6.0	8.7	14.9	0.165^b^

*BMI* Body mass index, *IQR* Interquartile range, *SGA* Small for gestational age, *HDP* Hypertension disorders of pregnancy, *PE* Preeclampsia, *GH* Gestational hypertension, *GDM* Gestational diabetes mellitus, *IRQ* Interquartile range

^a^*p* value, Jonckheere’s trend test

^b^*p* value, Extended Mantel–Haenszel Chi-square test

The results of the logistic regression analysis evaluating the effect of pre-pregnancy BMI on dystocia occurrence are reported in Table 3. The occurrence of dystocia was lowest in the G3 BMI group, and this group was used as a reference for the analyses. No significant risk factors for dystocia were identified for G1, G2, G4, and G5 BMI, after adjusting for several confounding factors in Models 2 and 3. G6 BMI was an independent risk factor for dystocia [OR, 3.20; 95% CI, 1.69–6.06], which persisted even after adjusting for several confounding factors in Models 2 and 3.

**Table 3:** Effect of body mass index before pregnancy on the occurrence of dystocia

BMI category (kg/m^2^)	< 18.5 (G1)	18.5 to < 20.0 (G2)	20.0 to < 23.0 (G3)	23.0 to < 25.0 (G4)	25.0 to < 30.0 (G5)	≥ 30.0 (G6)
Model1 OR (95% CI)	0.97 (0.53–1.77)	0.76 (0.43–1.33)	Ref	1.16 (0.60–-2.25)	1.74 (0.94–3.24)	3.20 (1.69–6.06)
Model 2 aOR (95% CI)	0.97 (0.52–1.80)	0.72 (0.40–1.28)	Ref	1.12 (0.56–2.22)	1.73 (0.91–3.23)	2.94 (1.50–5.77)
Model 3 aOR (95% CI)	0.97 (0.52–1.79)	0.72 (0.40–1.28)	Ref	1.12 (0.56–2.22)	1.72 (0.91–3.23)	2.95 (1.50–5.81)

*BMI* Body mass index, *OR* Odds ratio, *aOR* adjusted odds ratio, *CI* Confidence interval, *Ref* Reference group

Model 1 was calculated by univariate regression analysis, which calculated ORs

Model 2 adjusted for chronic maternal hypertension, maternal smoking, conception by assisted reproductive technology, maternal age (20–29 years as the reference), and uterine myoma

Model 3 adjusted as for Model 2 plus gestational diabetes mellitus

**Fig. 2:**
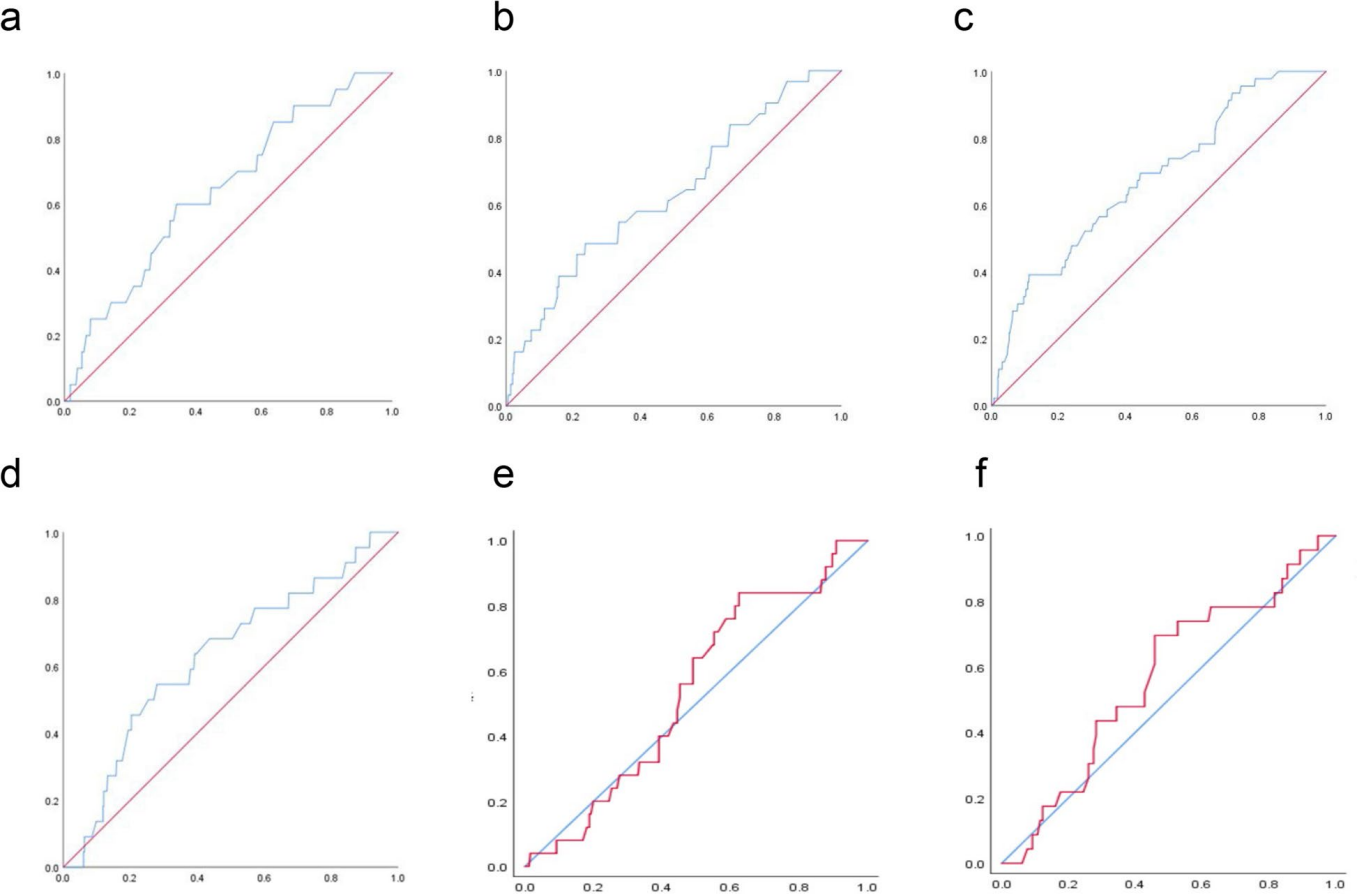
Receiver operating characteristic curve analysis of gestational weight gain predictive of dystocia by BMI category. Probability of weight gain during pregnancy associated with dystocia by BMI category; vertical axis, True Positive rate; horizontal axis: False Positive rate. **a** Receiver operating characteristic (ROC) curve for G1 (BMI < 18.5 kg/m^2^), with an area under the curve (AUC) of 0.641 (*P* = 0.019) and cutoff gestational weight gain of 11.5 kg (sensitivity, 0.500; specificity, 0.659). **b** ROC for G2 (BMI,18.5 to < 20.0 kg/m^2^); AUC, 0.634 (*P* = 0.010); cutoff, 12.3 kg (sensitivity, 0.484; specificity, 0.764). **c** ROC for G3 (BMI, 20.0 to < 23.0 kg/m^2^); AUC, 0.677 (*P* < 0.001); cutoff value, 14.3 kg (sensitivity, 0.391; specificity, 0.883). **d** ROC for G4 (BMI, 23.0 to < 25.0 kg/m^2^); AUC, 0.630 (*P* = 0.031); cutoff, 11.5 kg (sensitivity, 0.545; specificity, 0.621). **e** ROC for G5 (BMI, 25.0 to < 30.0 kg/m^2^); AUC, 0.540 (*P* = 0.446). **f** ROC for G6 (BMI ≥ 30.0 kg/m^2^); AUC, 0.567 (*P* = 0.291)

The ROC curve for dystocia according to weight gain during pregnancy for each BMI group is shown in Fig. 2. The AUC for G1 through G4 were 0.64, 0.63, 0.68, and 0.63, respectively (all *P* < 0.05). The cutoff values of gestational weight gain for each of these BMI groups were as follows: G1, 11.5 kg; G2, 12.3 kg; G3, 14.3 kg; and G4, 11.5 kg. Regarding G5 and G6, AUC was 0.54 (*P* = 0.45) and 0.57 (*P* = 0.29), respectively.

## Discussion

The occurrence of dystocia was highest in the G6 group (14.9%), which is consistent with a previous report [3], and lowest in the G3 group. Using G3 as the reference group, a pre-pregnancy BMI ≤ 25 kg/m^2^ (G6) increased the risk of dystocia, after adjusting for several confounding factors. Moreover, while the ROC analysis revealed a positive association between maternal gestational weight gain and the risk of dystocia for G1 through G4, with cutoff gestational weight gain associated with dystocia identified, this association did not hold in G5 and G6.

We noted that although previous studies have identified nullipara, fetal macrosomia, older maternal age, infertility treatment as risk factors for dystocia [4, 5, 26, 27], data on the association between pre-pregnancy BMI and dystocia or optimal gestational weight gain to reduce the risk of dystocia have not been comprehensively examined among nulliparous women.

It is widely accepted that pre-pregnancy BMI and gestational weight gain are associated with adverse obstetric outcomes, such as SGA, large for gestational age (LGA), CS, GDM, and HDPs [28]. Evidence that increased pre-pregnancy BMI is associated with abnormal labor has been established. Verdiales et al. reported that obese women whose BMI was > 35 kg/m^2^ had significantly higher frequency of arrest of cervical dilation than those whose BMI was < 26 kg/m^2^ (17.6 vs 5.2%; *p* = 0.005) [29]. Walsh et al. reported the potential influence of increasing maternal BMI at the first perinatal visit on intrapartum events among 3,158 nulliparous women in a developed country [9]. Using the BMI of nulliparous women at admission, Kominiarek et al. reported that higher BMI at admission and being nulliparous was strongly associated with delivery route among 124,389 term delivery pregnancies in the US [30]. Compared with previous studies, we focused on both BMI before pregnancy and gestational weight gain. Obesity in women during their reproductive years is of great concern in Western countries and one that is increasingly recognized in Japan [31]. In the Fukushima Prefecture, Japan, 12.7% of women have a BMI ≥ 25 kg/m^2^ at the start of their pregnancies [16], with the two Maternal–Fetal medical units included in our study having 17.0% more cases of obesity than that of the general population of women in Fukushima. As obesity increases the risk of CS, our finding that a high BMI before pregnancy increased the risk of dystocia, which occasionally required emergent CS, is reasonable. However, if obesity independently increases the risk of dystocia, maternal weight at the time of delivery, which reflects gestational weight gain, could affect the occurrence of dystocia, regardless of a lower pre-pregnancy BMI. Our findings suggest that although controlling pre-pregnancy BMI as a preconception counsel to reduce the risk of dystocia is advisable, providing guidance on the optimal gestational weight gain for women whose pre-pregnancy BMI < 25 kg/m^2^ is also essential.

Compared with the perinatal period, the preconception period provides a suitable opportunity for pregnant women to reconsider their lifestyle, with several efforts having focused on nutritional counseling for women of childbearing age and screening for nutritional status [32]. Furthermore, preconception nutritional counseling could assist in providing the motivation to alter food intake behavior during pregnancy and after delivery [24, 33–35], which could potentially affect neonatal neurodevelopment [36]. In Japan, the ratio of nullipara to mean maternal age at first delivery has increased in recent decades [20]. With this increase in maternal age, the number of pregnancies at risk of dystocia could increase. Therefore, the consensus about an gestational weight gain strategy to reduce the risk of dystocia will be required in the future.

The main strength of our study is that the data were derived from two tertiary care Maternal–Fetal medical units where all women who delivered were managed using approximately the same protocol [5, 37]. Furthermore, all participants in this study were Japanese women and, therefore, there were no effects of ethnic diversity on measured outcomes. This lack of ethnic diversity, however, also limits the generalizability of our findings. Other limitations of our study are as follows. We categorized women with a BMI ≥ 30.0 kg/m^2^ into one group. We noted that obesity has been further sub-classified as follows: class 1, BMI of 30 to < 35 kg/m^2^; class 2, BMI of 35 to < 40 kg/m^2^; and class 3, BMI ≥ 40 kg/m^2^ [38]. As obesity is a public health concern, a larger sample size to differentiate the risk between gestational weight gain and the risk of dystocia in both overweight and obese women would be warranted. In our multiple regression analyses we did not include several confounding factors previously identified as risk factors for dystocia (epidural analgesia, birth weight, and medical interventions, such as amniotomy) [4] as these were considered intermediate factors.

## Conclusion

There is little information on appropriate weight gain during pregnancy to reduce the risk of dystocia. We identified that although a BMI ≥ 30 kg/m^2^ independently increased the risk of dystocia, regardless of gestational weight gain, excessive gestational weight also increased the risk of dystocia among women with a pre-pregnancy BMI < 25 kg/m^2^. Our data provide an insight into the possible magnitude of the effect of preconceptional weight control and periconceptional weight gain on the risk of dystocia.

## Data Availability

The data supporting the findings of this study are available from the corresponding author upon reasonable request.

## Abbreviations

aOR: Adjusted odds ratio
AUC: Area under curve
ART: Assisted reproductive technology
BMI: Body mass index
CS: Cesarean section
CHT: Chronic hypertension
CI: Confidential interval
GDM: Gestational diabetes mellitus
GH: Gestational hypertension
GCT: Glucose challenge test
HDP: Hypertension disorders of pregnancy
OGTT: Oral glucose tolerance test
PE: Preeclampsia
RBG: Random blood glucose
ROC: Receiver operating characteristic curve
SGA: Small for gestational age
SD: Standard deviation
